# Frequency of Electrolyte Imbalance in Patients Presenting With Acute Stroke

**DOI:** 10.7759/cureus.18307

**Published:** 2021-09-27

**Authors:** Farah Mansoor, Jitesh Kumar, Navneet Kaur, Sandresh Sultan, Hamza Tahir, Anum Dilip, Faryal Khan, Narindar Kumar, Haya Khalid, Abdul Subhan Talpur

**Affiliations:** 1 Internal Medicine, Jinnah Sindh Medical University, Karachi, PAK; 2 Internal Medicine, Ghulam Muhammad Mahar Medical College, Sukkur, PAK; 3 Internal Medicine, Adesh Institute of Medical Sciences and Research, Buchu Kalan, IND; 4 Internal Medicine, Allama Iqbal Medical College, Lahore, PAK; 5 Internal Medicine, Bhitai Dental and Medical College, Mirpur Khas, PAK; 6 Internal Medicine, Liaquat University of Medical and Health Sciences, Jamshoro, PAK

**Keywords:** hemorrhagic stroke, ischemic stroke, stroke, hypokalemia, hyponatremia, electrolyte imbalance

## Abstract

Introduction

Electrolyte disturbances are commonly reported in acute stroke in studies conducted in the western world. Presently, the data available about the prevalence of electrolyte disturbance in patients with stroke are not sufficient, especially from developing countries. The purpose of our study is to determine the frequency of occurrence of electrolyte imbalance in patients presenting with acute stroke in a tertiary care hospital.

Methods

This descriptive cross-sectional study was conducted in the department of internal medicine and neurology in a tertiary care hospital, Pakistan, from December 2019 to March 2021. A total of 300 patients, aged between 30 and 70 years, with either ischemic or hemorrhagic stroke, as diagnosed on contrast tomography (CT) scan of the head or magnetic resonance imaging (MRI) of the brain, were enrolled in the study. The biochemical analysis of the stroke patients was done.

Results

Out of the 300 participants, 139 (46.3%) participants were from the ischemic stroke group while 161 (53.7%) were from the hemorrhagic stroke group. The mean sodium level was significantly lower in the ischemic group as compared to the hemorrhagic group (129.41 ± 3.12 mEq/L vs. 134.42 ± 3.46 mEq/L; p-value: <0.0001). Potassium level was significantly higher in the hemorrhagic group compared to the ischemic group (6.27 ± 1.12 mmol vs. 4.31 ± 0.71 mmol; p-value: <0.0001).

Conclusion

Patients coming to emergency with stroke should be screened immediately for electrolyte imbalance. Early identification of rapid imbalances of serum electrolytes may aid in prompt medical intervention and resultant improved outcomes in stroke patients. It is crucial that electrolyte imbalances in these patients are closely monitored to avoid any complications.

## Introduction

Ischemic infarction or hemorrhage in the brain leads to a state of non-convulsive focal neurological deficit of abrupt onset known as stroke or cerebrovascular accident (CVA). Out of all the neurological disorders, CVA is the most debilitating and is ranked as the third leading cause of death [1]. According to the World Health Organization (WHO), the mortality due to stroke accounts for approximately 85% in developing countries [2]. The incidence of stroke has declined in the western population during the last three decades. On the contrary, the burden of the disease in South Asian countries, such as India, Pakistan, Bangladesh, and Sri Lanka, is likely to rise [3]. The Pakistani data regarding the exact epidemiology are insufficient, yet stroke is found to be the most common reason for admission in neurology and medical wards [4].

Electrolyte disturbances are commonly found among other metabolic problems in patients with acute ischemic stroke. It is a potential cause of patient death unless corrected promptly. The disorders of sodium (Na) and potassium (K) balance are identified as the most common electrolyte abnormalities in patients with acute stroke [5]. Patients with hemorrhagic stroke present with symptoms like headache and vomiting, which in turn is a potential cause of electrolyte imbalance. This disturbance in the electrolyte balance is due to deranged secretion of antidiuretic hormones (ADHs), rise in the levels of atrial and brain natriuretic peptides, and inappropriate fluid input and output, causing complications like seizures and death [6]. Therefore, in order to prevent morbidity and mortality in CVA patients, early diagnosis of electrolyte imbalance is important [7].

Presently, the data available about the prevalence of electrolyte disturbance in patients with stroke are not enough especially from developing countries. The purpose of our study is to determine the frequency of occurrence of electrolyte imbalance in patients presenting with acute stroke in a tertiary care hospital.

## Materials and methods

This study is a descriptive cross-sectional study, conducted in the department of internal medicine and neurology in a tertiary care hospital, Pakistan, from December 2019 to March 2021. A total of 300 patients, aged between 30 and 70 years, with either ischemic or hemorrhagic stroke, as seen on contrast tomography (CT) scan of the head or magnetic resonance imaging (MRI) of the brain, were enrolled in the study. Diagnosis was made by a neurologist. Consecutive convenient non-probability sampling was used. Before enrollment, approval from the ethical review board was taken from Jinnah Sindh Medical University (JSMU/2019/IRB/64). Patients with chronic kidney disease were excluded from the study.

First, informed consent was taken either from the patients or from the attendants in cases where the patient was unconscious or not responsive. After collecting the demographics such as age and gender via self-structured questionnaire, the tests performed included CT scan of the head or MRI brain, depending on the availability of either, complete blood count, blood glucose, urea, creatinine, liver function tests, chest x-ray, and serum electrolytes, i.e. Na, K, chloride (Cl), and calcium (Ca). Management was stated immediately after collection of blood sample. For every patient with stroke, the biochemical analysis was done in the same laboratory in order to maintain uniformity of the test results.

The Statistical Package for Social Sciences, version 22.0 (SPSS, IBM Corporation, Armonk, NY, USA) was used to analyze the data. For continuous variables, the mean and standard deviation were used, while categorical variables were represented by percentages and frequencies. Chi-square and dependent t-test were applied to compare the categorical and numerical data, as appropriate. A p-value of less than 0.05 was considered statistically significant.

## Results

Out of the 300 participants, 139 (46.3%) participants were from the ischemic stroke group while 161 (53.7%) were from the hemorrhagic stroke group. Patients with hemorrhagic stroke were younger compared to the patients with ischemic stroke (49 ± 14 vs. 64 ± 9; p-value: <0.0001). The systolic blood pressure was significantly higher in the hemorrhagic stroke group compared to the ischemic stroke group (167.36 ± 37.51 mmHg vs. 139.34 ± 31.82 mmHg; p-value: <0.001). Random blood sugar was significantly higher in the ischemic stroke group compared to their counterparts (176.21 ± 92.31 vs. 131.20 ± 46.30; p-value: <0.0001) (Table 1).

**Table 1:** Characteristics of the study participants ALP: alkaline phosphatase; ALT: alanine transaminase; BUN: blood urea nitrogen; CBC: complete blood count; DBP: diastolic blood pressure; Hb: hemoglobin; IU/L: international units per liter; mg/dL: milligram per deciliter; mmHg: millimeters of mercury; RBS: random blood sugar; SBP: systolic blood pressure; WBC: white blood count

Characteristics	Ischemic stroke (n = 139)	Hemorrhagic stroke (n = 161)	p-Value
Age (years)	64 ± 9	49 ± 14	<0.0001
Male			
SBP (mmHg)	139.34 ± 31.82	167.36 ± 37.51	<0.0001
DBP (mmHg)	85.42 ± 15.11	91.77 ± 17.84	0.0011
Pulse rate (per minute)	81.29 ± 11.51	84.50 ± 10.21	0.0110
CBC			
Hb (%)	12.91 ± 2.16	13.01 ± 2.01	0.6784
WBC (per microliter)	18128.00 ± 2187.59	16700.00 ± 3608.86	0.0001
Platelet (per microliter)	246080.0 ± 20162.44	26000.0 ± 192251.9	<0.0001
Liver enzymes			
ALT (IU/L)	136.51 ± 22.16	140.72 ± 25.23	0.1285
ALP (IU/L)	121.16 ± 62.79	133.69 ± 45.91	0.0475
Bilirubin (IU/L)	0.62 ± 0.31	0.82 ± 0.37	<0.0001
Other values			
Creatinine (mg/dL)	1.02 ± 0.49	0.99 ± 0.47	0.5892
BUN	60.39 ± 36.32	49.71 ± 24.15	0.0026
RBS (mg/dL)	176.21 ± 92.31	131.20 ± 46.30	<0.0001

The Na level was significantly lower in the ischemic group as compared to the hemorrhagic group (129.41 ± 3.12 mEq/L vs. 134.42 ± 3.46 mEq/L; p-value: <0.0001). The K level was significantly higher in the hemorrhagic group compared to the ischemic group (6.27 ± 1.12 mmol vs. 4.31 ± 0.71 mmol; p-value: <0.0001). No significant difference in Cl levels was found (Table 2).

**Table 2:** Comparison of serum electrolyte levels in both groups mEq/L: milliequivalents per liter; mmol/L: millimoles per liter

Electrolytes	Ischemic stroke (n = 139)	Hemorrhagic stroke (n = 161)	p-Value
Sodium (mmol/L)	129.41 ± 3.12	134.42 ± 3.46	<0.0001
Potassium (mmol/L)	4.31 ± 0.71	6.27 ± 1.12	<0.0001
Chloride (mmol/L)	99.21 ± 5.51	98.98 ± 4.91	0.7025

## Discussion

The results of our study demonstrated that ischemic stroke was more common in younger participants. Patients with hemorrhagic stroke had higher blood pressure. Moreover, the ischemic group had a lower concentration of Na while the hemorrhagic group had a higher concentration of K. The results of our study are in line with other studies. Studies conducted by Rodrigues et al. and Soiza et al. reported that patients with reduced Na levels or hyponatremia were more inclined toward stroke severity and risk for mortality [8,9]. On the other hand, other researches conducted by Christensen et al. and Farahmand et al. demonstrated that increased venous serum Na concentrations were associated with a higher incidence of stroke and worsening of the neurological conditions [10,11].

Hyponatremia occurs either due to the retention of water or loss of Na [12]. This occurs due to the impairment of renal excretion of water; however, this impairment does not occur in patients whose water intake is approximately 10-15 L/day [12]. For the development and maintenance of hyponatremia, the suppression of ADH secretion is essential for the excretion of any water load and low plasma osmolality [12]. Most causes of hyponatremia in stroke are related to an absolute or relative excess of ADH, most likely due to the syndrome of inappropriate ADH secretion (SIADH) or the depletion of effective circulating volume [12].

Hyponatremia is classified as pseudo-, true, and translocational hyponatremia. Conditions such as multiple myeloma cause hyperproteinemia and/or increased triglyceride levels, which result in pseudo hyponatremia (normo‑osmolal) [13]. On the other hand, a higher serum osmolality, secondary to mannitol or glucose, results in translocational (hyperosmolal) or hypertonic or redistributive hyponatremia [14]. The reduction in the osmotically active solutes in the serum causes true (hypoosmolal) hyponatremia, which is further subclassified as hyper-, hypo-, and euvolemic [15,16]. Euvolemic contributes to 60% of all cases of hyponatremia. SIADH is known as the most common cause of euvolemic hyponatremia. Hypervolemic hyponatremia mostly occurs in cases of congestive heart failure and liver cirrhosis, nephrotic syndrome, and chronic kidney disease [13].

The study has the following limitations. First, the study was conducted in a single institute, hence the sample size and diversity were limited. Secondly, since it was a cross-sectional study, the definite association could not be established. Thirdly, impact of electrolyte imbalance on outcome of patients was not measured. Therefore, more large-scale studies are needed for larger and diverse sample size and to effectively establish the relationship between the two. 

## Conclusions

In our study, Na levels were significantly lower in patients with ischemic stroke, whereas K levels were significantly higher in patients with hemorrhagic stroke. Patients coming to emergency with stroke should be screened immediately for electrolyte imbalance. Early identification of rapid imbalances of serum electrolytes may aid in prompt medical intervention and resultant improved outcomes in stroke patients. Severe imbalances may cause deterioration of the neurological status of these patients. Hence, it is crucial that electrolyte imbalances in these patients are closely monitored to avoid any complications.
